# Inflammatory Immune-Associated eRNA: Mechanisms, Functions and Therapeutic Prospects

**DOI:** 10.3389/fimmu.2022.849451

**Published:** 2022-04-19

**Authors:** Lilin Wan, Wenchao Li, Yuan Meng, Yue Hou, Ming Chen, Bin Xu

**Affiliations:** ^1^ Medical School, Southeast University, Nanjing, China; ^2^ Department of Urology, Affiliated Zhongda Hospital of Southeast University, Nanjing, China; ^3^ Department of Urology, Nanjing Lishui District People’s Hospital, Zhongda Hospital, Southeast University, Nanjing, China; ^4^ Key Laboratory of Biomedical Information Engineering of Ministry of Education, Biomedical Informatics and Genomics Center, School of Life Science and Technology, Xi’an Jiaotong University, Xi’an, China

**Keywords:** eRNA, immune inflammatory, enhancer transcription events, cancers, therapeutic prospects

## Abstract

The rapid development of multiple high-throughput sequencing technologies has made it possible to explore the critical roles and mechanisms of functional enhancers and enhancer RNAs (eRNAs). The inflammatory immune response, as a fundamental pathological process in infectious diseases, cancers and immune disorders, coordinates the balance between the internal and external environment of the organism. It has been shown that both active enhancers and intranuclear eRNAs are preferentially expressed over inflammation-related genes in response to inflammatory stimuli, suggesting that enhancer transcription events and their products influence the expression and function of inflammatory genes. Therefore, in this review, we summarize and discuss the relevant inflammatory roles and regulatory mechanisms of eRNAs in inflammatory immune cells, non-inflammatory immune cells, inflammatory immune diseases and tumors, and explore the potential therapeutic effects of enhancer inhibitors affecting eRNA production for diseases with inflammatory immune responses.

## Highlights

This review summarized the relevant roles of eRNAs in inflammatory immune functions, mechanisms and therapies, and explored the research directions and target therapy prospects for inflammatory immune-related eRNAs.

## Introduction

The rapid development of high-throughput sequencing technologies has made it possible to identify potential functional regulatory elements. To date, studies have identified numerous regulatory elements, such as enhancers, promoters and silencers (1).

**Figure 1:**
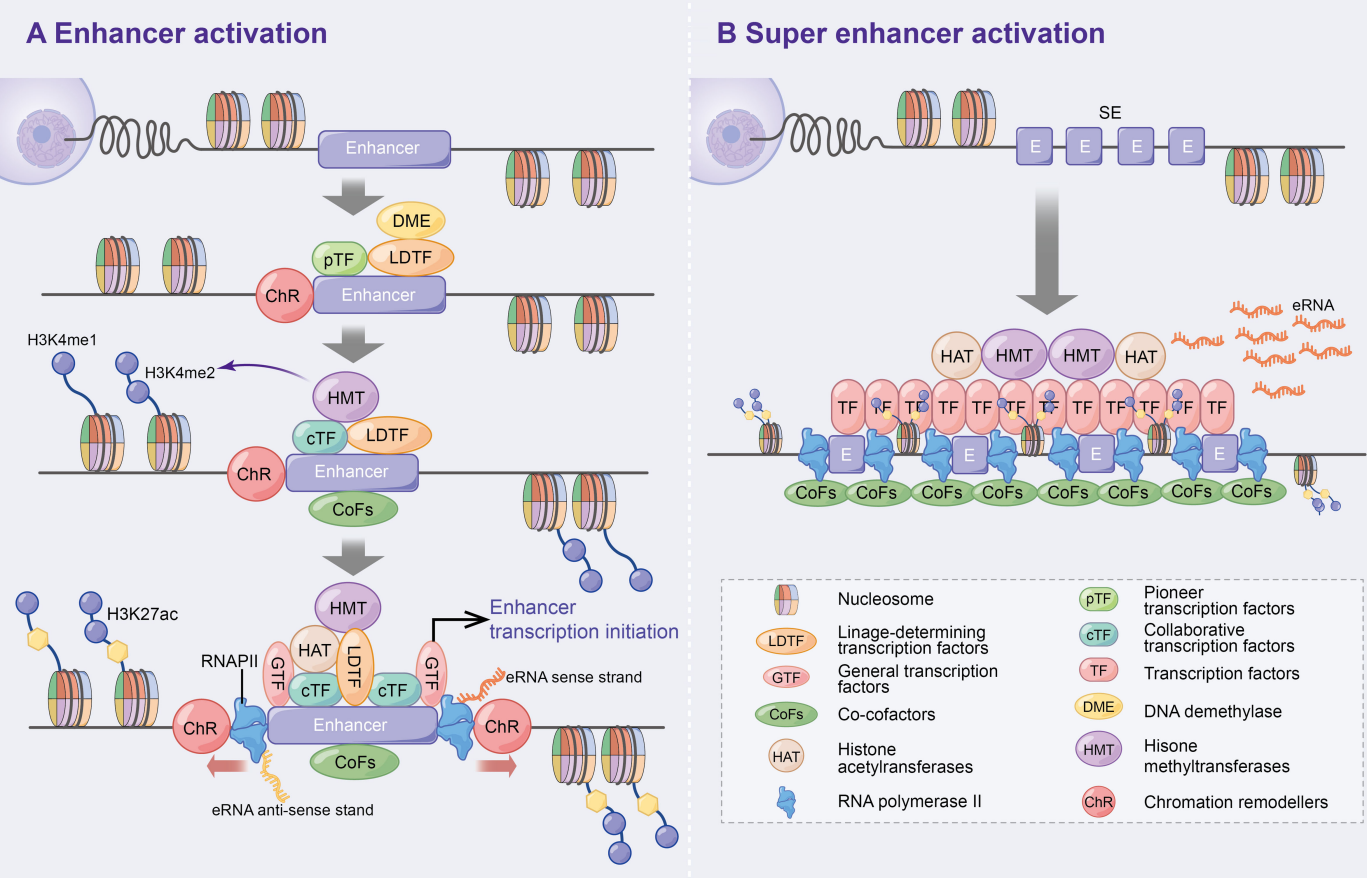
Activation mechanism of enhancers and super enhancer. **(A)** Enhancer activation. Multifactorial stimulation causes pTFs binding to nucleosomes leading to ChR and further recruitment of LDTFs, cTFs and CoFs that assist enhancer activation and histone methylation. On this basis, HAT, GTFs and RNAPII are recruited and ultimately contribute to enhancer transcription bi-directionally. In addition, DME are recruited to regulate DNA methylation and p300 status at the initial stage of enhancer activation. **(B)** Super enhancers are large clusters of enhancers with high levels of cell-specific transcription factors and cofactors that can ultimately express higher levels of eRNAs.

Enhancers, which are distal regulatory DNA sequences, are approximately 500–2000 bp in length and function independently of orientation (2, 3). Since the discovery of the first enhancer SV40 by Banerji J et al. in 1981 (4), the specific function and mechanism of enhancers have been extensively explored. Numerous studies have revealed that pioneer transcription factors enhance nucleosome DNA to generate open chromatin (5), which promotes the recruitment of lineage-determining transcription factors (LDTFs) to maintain the activated state of enhancers (6). Subsequently, collaborative transcription factors (cTFs) and co-cofactors (CoFs), such as histone methyltransferases, are recruited, which promote histone 3 lysine 4 monomethylation (H3K4me1) and dimethylation (H3K4me2) (6–10). Following this, histone acetyltransferases (HAT) [e.g. CBP/p300 to promote H3K27 acetylation (ac)] (10, 11), general transcription factors (GTFs) (12) and RNA polymerase II (RNAPII) (3, 13) are further recruited to initiate enhancer-associated bidirectional transcription. Additionally, DNA demethylase (DME) is recruited during the initial phase of enhancer activation to regulate dynamic DNA methylation and p300 binding (14, 15) (**Figure 1A**). Consistent with previous studies, the above study suggests that enhancers positively regulate spatiotemporal gene expression (4, 16, 17).

Super enhancers (SEs) were first found in mouse embryonic stem cells and tumour cell lines (18, 19). Compared to typical enhancers, SEs consist of large enhancer clusters with longer genomic regions, higher cell-specific transcription factor (TF) levels (e.g. Oct4, Sox2, Nanog, Eseeb and Klf4) (18, 20–22), CoFs (e.g. BRD4, Mediator and CDK9) (21), higher H3K4me1 expressions and H3K27ac modifications (23, 24). These characteristics allow SEs to highly express super enhancer derived super RNAs (seRNAs) (**Figure 1B**). Therefore, SEs have been speculated to be the key determinants of cell identity and fate (18, 23), and thereby lead to increased disease susceptibility when mutated (20, 23).

eRNA, as the enhancers transcription product, belongs to the class of non-coding RNA (ncRNA). Based on differences in function and size, ncRNAs are classified as long non coding RNA (lncRNA), promoter associated RNA (paRNA), enhancer RNA (eRNA), small RNA (sRNA), PIWI interacting RNA (piRNA), small nucleolar RNA (snoRNA), Small nuclear RNA (snRNA), ribosomal RNA (rRNA), micro RNA (miRNA) etc (25–28). Similarly, they are generated in different parts of the genome and can be obtained by unidirectional or bidirectional transcription, with variable stability and longevity (26–28). The section will briefly describe the differences and commonalities between eRNA and lncRNA. eRNAs are similar in length to lncRNAs, about 0.1-9 Kb, but are divided into two classes of ncRNAs by their associated histone profiles (29). In the 2010 study, Orom UA et al. (30) found that lncRNAs can regulate the expression of neighboring genes. Subsequently, this type of lncRNA was revealed to be an enhancer of some protein-coding genes, but its chromatin characteristics differ from typical enhancers and it is usually polyadenylated and does not have the characteristics of bidirectional transcription of eRNA. Further studies suggest that such lncRNAs carry intermediary complexes to neighboring genes *via* chromatin loops to influence their expression (30, 31). However, most eRNA transcripts are 5’ cap-shaped, unspliced, unpolyadenylated and have a short half-life, but are also dynamically regulated with signaling and correlate with increased expression of neighboring genes (32).

To date, numerous reviews have summarized the mechanisms and potential functions of enhancers, SEs and their transcription products (eRNAs) in tumours (33–36). Moreover, the expansion of the study dimension revealed that enhancers, SEs and their eRNAs were immediately altered in response to inflammatory stimuli, leading to abnormal inflammatory gene expression. This observation strongly suggests their involvement in biological processes, such as inflammation, immunity and neurodegeneration (24, 37–39). Yoshiki et al. (21) have summarized the potential role of SEs in inflammatory gene transcription.

**Figure 2:**
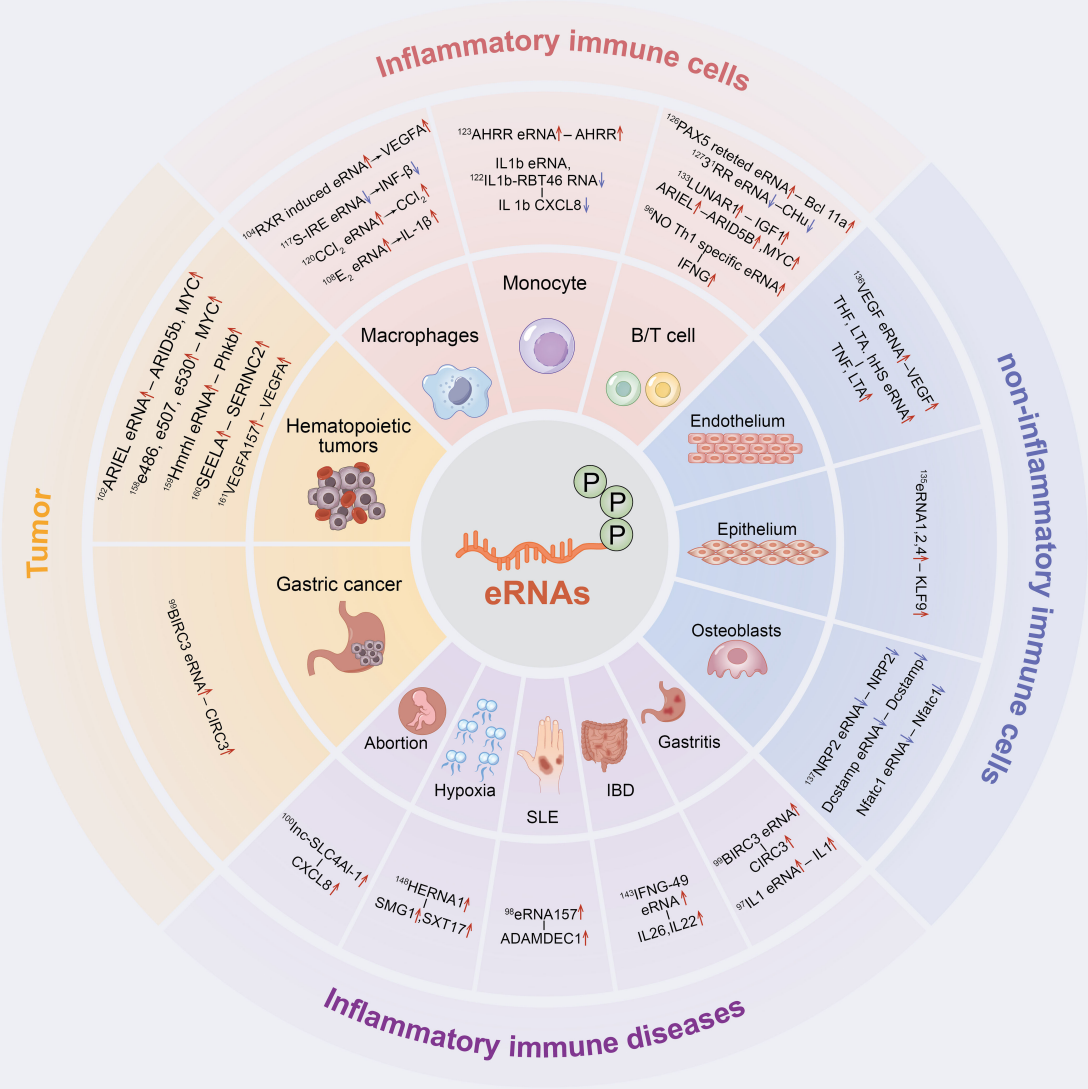
The inflammatory immune role of eRNAs in cells and diseases. eRNAs have a significant contribution in inflammatory immune cells, non-inflammatory immune cells, inflammatory immune diseases and tumor inflammatory alterations.

However, to the best of our knowledge, the potential mechanisms and roles of eRNAs in inflammatory immune diseases are yet to be reviewed. This study aims to summarize the potential roles of eRNAs in inflammatory immune cells, non-inflammatory immune cells, inflammatory immune diseases and tumour inflammatory alterations, and thereby elucidate the relevance of eRNAs in inflammatory immunity (**Figure 2**). Additionally, the potential role of eRNAs as novel therapeutic targets and prognostic biomarkers for diseases with inflammatory immune alterations are explored.

## Results

### Mechanism of Enhancer Transcription and eRNA in Regulating Target Genes

#### Effect of Enhancer Transcription on Their Products and Target Genes

The mechanism of enhancer activation has been discussed in the Introduction (**Figure 1A**); therefore, the regulation of downstream target genes by enhancer transcription (including initiation, elongation, termination and degradation) will be discussed in this section.

**Figure 3:**
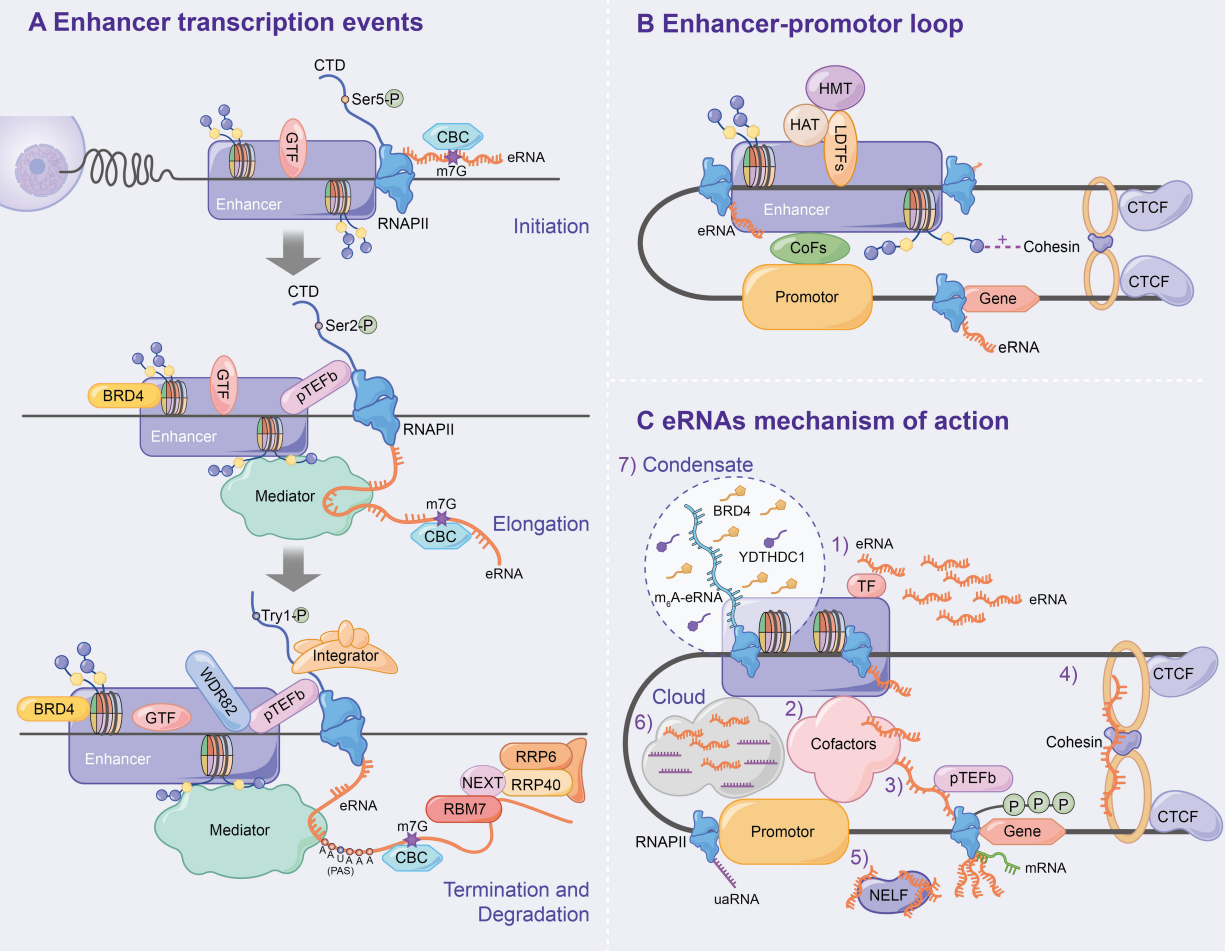
Mechanism of enhancer transcription and eRNAs to regulate target genes. **(A)** Following enhancer activation, GTFs and serine 5-phosphorylated form of RNAPII (Ser5p) are recruited, which leads CBC to binding to eRNA *via* m7G, ultimately initiating enhancer transcription. During the enhancer elongation, Ser2p is hypoenriched. pTEFb, BRD4 and the eRNAs being transcribed all affect transcription elongation. Subsequently, the nascent eRNAs interacts with RNAPII CTD to terminate transcription. In addition, the integrator, WDR82 and Tyr1p assist in correct enhancer transcription termination. Finally, the NEXT complex mediates the degradation of eRNAs. **(B)** Chromatin loops are generated under the influence of LDTFs, histone methylation or acetylation, cohesin-CTCF complex and interphase chromosome topologically associated domain, and ultimately activate target gene transcription. **(C)** Specific mechanisms have been demonstrated for eRNAs to affect enhancer activity, E-P loop formation, and transcription of downstream target genes.

GTFs (e.g. TBP) and the serine 5-phosphorylated form of RNAPII (Ser5p was engaged in RNA capping mechanism) have been detected in the enhancer region in the transcription of lncRNAs or mRNAs (40). Moreover, global nuclear run-on sequencing (GRO-seq) and cap analysis gene expression (CAGE) results further suggest that eRNAs are capped (5′ end 7-methylguanosine (m7G) cap facilitates cap-binding complex (CBC) recruitment), with significant similarities in DNA sequences, core promoter elements and nucleosome spacing at enhancer and promoter transcription start sites (TSSs) (41–43), and promote bidirectional transcription (29, 32, 42, 44), thus indicating that the rules of transcription initiation apply to enhancers and promoters. Similar to promoters, the direction of enhancer transcription is also determined by the ratio of the relative density of polyA cleavage sites (PASs) and the U1 splice motif downstream of the TSSs, which affects the production of eRNAs initiated by RNAPII elongation (41, 42, 44–46) (**Figure 3A**).

The elongation, termination and RNA processing stages highlight transcriptional differences between enhancers and promoters. Ser2p, a form of RNAPII that is involved in elongation, is characterized by low enrichment, which results in low H3K36me3 levels and affects enhancer transcription elongation (40, 47, 48). The enhancer transcription elongation phase was found to be partially impacted by the overlapping mechanisms of the coding genes, including positive transcription elongation factor-b complex (pTEFb) and bromodomain-containing protein 4 (BRD4, which is recruited by H3K27ac) (10–12). Additionally, eRNAs undergoing transcription bind to the mediator complex and affect transcription elongation (18, 31, 49) (**Figure 3A**).

Numerous studies have found that PAS-mediated early termination regulates eRNA stability, and RNA exosomes degrade eRNAs. A study by Lai et al. confirmed that the integrator, which interacts with the RNAPII carboxy-terminal domain (RNAPII CTD), is an important regulator of eRNA termination (50) that functions after the nascent eRNAs have the PAS (AAUAAA) (42, 44, 50, 51). Integrator subunit deletion decreases eRNA levels and increases enhancer transcription activity, indicating the disruption of eRNA termination (50). Additionally, WD repeat-containing protein 82 (WDR82) acts as an adaptor protein that targets SET1 H3K4 methyltransferase, affecting enhancer transcription termination (52). Interestingly, Tyrosine 1-phosphorylated form of RNAPII (Tyr1p) was highly enriched in the active enhancer and PROMPT (upstream transcripts of the promoter) regions, whereas it was slightly enriched in the gene promoter region (53, 54). Moreover, Yeast Tyr1p was shown to be associated with eRNA termination (55, 56). In addition, under the mediation of the trimeric nuclear exosome targeting (NEXT) complex, wherein RBM7 directly binds to eRNAs, exosome component 10 (EXOSC10/RRP6) and EXOSC3 (RP40) were reported to be responsible for the final degradation of eRNAs (57). The recruitment of NEXT may also be facilitated by the CBC (57, 58) (**Figure 3A**).

The chromatin loop, including enhancer-promoter loop or E-P loop, as the optimal mediator of target gene expression, was found to play an important role in enhancer transcription and eRNA production *via* enhancer-promoter proximity facilitation (59–61) (**Figure 3B**). Since β-globin loci discovery, enhancer-promoter-mediated chromatin loops have been detected at multiple enhancer locus control regions (LCRs) interacting with target genes through chromosome conformation capture (3C) (62, 63). LDTFs [e.g. NF-E2 (64)] were found to directly anchor E-P loop regions to recruit cTFs, CoFs or histone-modifying enzymes, which affects E-P loop formation and enhancer activation (64–68). H3K4me1 improves interchromatin interactions between enhancers and promoters by promoting chromatin recruitment to the cohesin complex (69), whereas H3K27ac affects enhancer-promoter activity by destabilising nucleosomes or recruiting H3K27ac-binding proteins (70). CCCTC-binding factor (CTCF), a highly conserved zinc finger protein, is co-localized with cohesin and has an independent cohesion binding site (71, 72). The cohesin-CTCF complex creates repressor regions (blocking chromatin repressor region diffusions and blocking enhancer activity) and active regions (promoting enhancer to promoter proximity) to regulate chromatin homeostasis, which contributes to the formation and stabilization of long-term chromatin interactions, thus, affecting E-P loop formation and transcription progression (73–78). Using molecular dynamics models, Dusan et al. (79) demonstrated that transcription-induced superhelix in the interphase chromosome topologically associated domain (TAD) formation is the driving force for chromatin loop extrusion, which stimulated enhancer-promoter contacts and activates mRNA transcription in a given TAD.

#### Effect of eRNA on Target Genes

eRNAs, a transcription product of enhancers, affect enhancer activity, E-P loop formation and downstream target gene transcription. Numerous studies have found that eRNA has several mechanisms as follows (**Figure 3C**): 1) eRNA positively regulates enhancer transcription and stabilizes gene expression by binding to TFs (e.g. YY1) (12); 2) eRNA promotes active enhancer acetylation by recruiting Cofactors (e.g. CBP/300) (80) and interacts with various other Cofactors Complexes [e.g. RAD21 and heterogeneous nuclear ribonucleoprotein U (hnRNPU)] to stabilize the E-P loop and regulate target gene expression (81–83); 3) eRNAs directly induce E-P loop formation and histone modification by linking p300 (as Cofactors) and RNAP II (whose RNAP II are present on the enhancer and promoter, respectively) (84); 4) eRNA stabilizes the E-P loop structure by absorbing cohesin and subsequently regulating gene expression (85); 5) eRNA binds to the paused RNAPII by competing with mRNA, which allows the negative elongation factor (NELF) complex to separate from RNAPII and bind to eRNA, leading to RNAPII phosphorylation and the positive transcription elongation factor b (P-TEFb) recruitment and allowing RNAPII to enter the transcription elongation phase and produce mRNAs (86); 6) Cai et al. proposed for the first time that enhancers and promoters form a local molecular cloud during transcription, which consists of eRNA and uaRNA (eRNA promoter transcription analogues), bringing enhancers and promoters closer to facilitate E-P loop formation (87); 7) The recent studies have confirmed the prevalence of m6A modification in nascent eRNA, which recruits the nuclear m6A reader YTHDC1 to form a liquid-like condensate in a manner dependent on its C terminus intrinsically disordered region and arginine residues. Subsequently, the m6A-eRNA/YTHDC1 condensate co-mixes with and facilitates the formation of BRD4 coactivator condensate, ultimately activating the gene (88); 8) Aguilo et al. (89) demonstrated that NOP2/Sun RNA methyltransferase (NSUN7) can deposit 5-cytosine methylation on eRNA, which affects eRNA stability and regulates enhancer transcription; 9) eRNA regulates gene expression by modulating E-P interactions and higher chromatin structure topology (9).

#### Potential Regulatory Mechanisms of eRNA in Inflammatory Immunity

Acute and chronic inflammation is the adaptive response to the internal environment and external stimuli, and the underlying pathological events leading to atherosclerosis, cancer, infectious diseases and immune disorders. The production of active enhancers and eRNA production in the nucleus precedes inflammatory gene expression in response to lipopolysaccharide (LPS) stimuli (90–92). Comprehensive studies of eRNA inflammatory immune-related mechanisms have revealed that the binding of LDTF (e.g. PU.1/T-bet/AP-1 and C/EBP) to the enhancer regions under inflammatory mediator stimulation promotes nucleosome remodeling to create nucleosome-free regions and H3K4me1/2 modification and recruits RNAPII (13, 80, 93–96). Subsequently, inflammation-associated signal-dependent TF NFκB-p65/p50 binds to the enhancer region and leads to histone H3K27ac modification and eRNA transcriptional production (10, 13, 93, 97–100). The generated eRNAs assist the tight junctions of the mediator complexes (p300-BRD and pTEFb-MLL) using enhancer-promoter interactions to stabilize the E-P loop (13, 97, 99, 101, 102). Conversely, eRNAs coordinate cohesin-CTCF loop formation to promote chromosome cyclisation (13, 79, 103, 104). Therefore, eRNA regulates enhancers and downstream target gene transcription through the aforementioned methods, and the mRNA produced is translocated outside the nucleus and generates associated inflammatory factors (such as INF-β, IL-1β, TNF and CXCL8), which affect inflammatory immune cell response and related pathological responses.

In the following sections, we will summarize in detail the role of eRNAs in inflammatory immune cells, non-inflammatory immune cells, inflammatory immune diseases and tumors as well as their related mechanisms (**Figure 2**).

### eRNA in Inflammatory Immune Cells

LPS, a major component of the outer wall of the gram-negative bacterial cell wall, activate mononuclear macrophages, lymphocyte, endothelial cells and epithelial cells through cellular signaling systems. Various cytokines and inflammatory mediators are synthesized and released in this process, which induces an inflammatory immune response (105–107). Nasun et al. (91) were the first to use toll-like receptor 4 signaling in macrophages as a model to clarify that SE-associated eRNA transcription is dynamically induced in most key genes driving innate immunity and inflammation using GRO-seq. Subsequently, CAGE data further verified that enhancer transcription preceded the target gene activation in monocytes under LPS stimulation (41, 90). Recently, Ma et al. (92) used simultaneous high-throughput ATAC and RNA expression with sequencing (SHARE-seq) to identify domains of regulatory chromatin (DORCs) that significantly overlap with SEs, which showed both chromatin accessibility and enhancer lineage-priming precede gene expression. This suggests that altered chromatin accessibility could be a pre-condition for cell lineage formation. Therefore, current studies have shown that both enhancer transcription and eRNA precede the transcriptional induction of inflammatory genes in the LPS response (90–92) and eRNA links enhancer activity to inflammatory gene expression through CBP-mediated H3K27 acetylation (80). Conversely, the plasticity of mature immune cells attracts us to explore the potential function of eRNAs in inflammatory immune cells (108, 109) (**Table 1**).

**Table 1:** List of eRNAs involved in inflammatory immune cells.

Cell type	Affected cell	Enhancer or SE	eRNA	Regulated gene	Mechanism	Function	Reference
**Macrophages**	Macrophages	Vegfa and Tgm2	RXR-induced eRNA	Angiogenic genes (Vegfa)	Stabilization of RXR-induced E-P loop	Induce angiogenesis	(110)
	Macrophages	IFN-β-specific enhancer	S-IRE1 eRNA	IFN-β and IFNB1	Interaction of Kdm6a with MLL4	Promote production of inflammatory factors	(111)
	Macrophages	Enhancer2	E2 eRNA	IL-1β	Mediated by PU.1 and NF-kB	Promote production of inflammatory factors	(112)
	Macrophages	Ccl2 enhancer	Ccl2 eRNA	Ccl2 (MCP-1)	(GPS2 and SMRT)-eRNA-CCL2 regulatory axis	Promote inflammation and insulin resistance	(113)
**Monocyte**	Primary monocytes	Enhancer	IL1b eRNA IL1b-RBT46 eRNA	IL1b CXCL8	Mediation of the pro-inflammatory transcription factor NF-kB	Promotes the release of pro-inflammatory factors	(114)
	CD14+ monocyte	AHRR enhancer	AHRR eRNA	AHRR	Affection of pol II recruitment in transcription	Influence cell type-specific AHRR	(115)
	THP-1 monocytes	hHS-8 enhancer	TNF eRNA, LTA eRNA, hHS eRNA	TNF, LTA	No	Influence monocyte inflammatory immunity	(116)
**B lymphocytes**	B cell	No	LNCGme00432, 00344, 00345	Bcl11a	No	Cause malignant development of B cells	(117)
	B cell	3’RR SE Eμ enhancer	3’RR eRNA	CHμ	Regulation of SHM and conventional CSR	Affect the B-cell maturation process	(118)
**T lymphocytes**	T-ALL cells	No	LUNAR1	IGF1R	Mediation of notch signaling pathway	Maintain the malignant progression of T-ALL	(119)
	T-ALL cells	ARID5B enhancer	ARIEL	ARID5B MYC	Regulation of the TAL1-induced transcriptional program	Accelerate T-cell malignant progression	(97)
	T cells	T-bet SE	eRNA	IFNG	Mediation of NF-kB	Regulation of Th cell differentiation	(91)
	CD4+ T cell	hHS-8 enhancer	TNF eRNA. LTA eRNA, hHS eRNA	TNF LTA	No	Affect T-cell pathological changes	(116)

#### eRNA in Macrophages

Macrophages, the key cells that maintain the tissue’s internal environment and regulate the inflammatory immune response, perform tissue-specific functions and defend against infections (91, 120). Numerous studies have suggested the presence of PU.1 TFs in the active enhancer region of macrophages, which identify chromatin DNA recognition motifs and C/EBP, thereby creating nucleosome-free regions and undergoing histone tail H3K4me1/2 modifications (10, 13, 29, 54, 93, 95, 110–113, 121). Subsequently, similar signal-dependent TFs, p65 and NFκB, bind to the enhancer region under LPS stimulation, resulting in histone H3K27ac modifications and eRNA transcriptional generation (10, 93).

After activation of the RXR signaling pathway, the RXR-induced eRNAs were detected on Vegfa and Tgm2 enhancers, with studies showing that the eRNAs maintained macrophage angiogenic activity *via* enhancer interactions (104). In 2018, a study by Bence et al. revealed that macrophages under multiple IL-4 stimulations showed increased IL-4-sensitive (Arg1 and Hbegf) and RSG/IL-4-sensitive (Tgm2) eRNA expressions, leading to active STAT6 recruitment, which induced macrophage phenotypic changes by affecting the nuclear receptor PPAR (122). Meanwhile, Zsolt et al. (111) revealed that eRNA expression levels in IL4-STAT6-mediated responses correlated with the enrichment of RNAPII-Ser5p and RNAPII-Ser2p and levels of the inhibitory and activating factor STAT6 locus H3K27ac, which reduced macrophage responsiveness to LPS and suppressed inflammatory responses, including inflammatory vesicle activation, IL-1b production and pyroptosis. Further studies have clarified that Kdm6a, a demethylase, not only promotes macrophage IL-6 expression through promoter H3K27me3 demethylation but also interacts with MLL4 to increase IFN-β-specific eRNA S-IRE1, thereby promoting IFN-β transcription in macrophages (114). Ha et al. (115) suggested that the PU.1-mediated E2 eRNA (E_2_ is a potential regulatory element of approximately 10 kbp that is located upstream of the TSS) in macrophages is essential for IL-1β mRNA transcription, which might influence the macrophage-assisted regulation of disease states, such as endotoxic shock, sepsis and infection. Additionally, Oishi et al. (116) found that RevErb expression regulated by Bmal1 was repressed in Arntl^-/-^ macrophages, with further studies revealing that RevErbs repressed eRNA transcription by recruiting the NCoR- histone deacetylase3 (HDAC) repressor complex and increasing enhancer H3K27 acetylation, thereby regulating enhancer epigenetic state to control macrophage inflammatory response (43, 116). Huang et al. (123) used inflammatory macrophage activation models to demonstrate that the inflammation activation-associated corepressor (GPS2 and SMRT)-eRNA-CCL2 regulatory axis, in addition to finding that LNA-targeted Ccl2 enhancer E-transcribed eRNA in white adipose tissue macrophages of obese (ob/ob) mice, can partially reverse meta inflammation and insulin resistance.

Therefore, macrophages activate intranuclear enhancer transcription and eRNA production in response to inflammatory stimuli, which regulates inflammatory factors and chemokine release and affects macrophage polarisation. However, the specific mechanisms of inflammatory genotypic and phenotypic changes by eRNA remain unclear and require further experimental exploration.

#### eRNA in Monocytes

Circulating monocytes, innate immune response cells, prevent infection by rapidly removing invading pathogens. Heward (117) and IIott (118) reported in the same year that monocytes are differentially expressed with large amounts of eRNAs in response to LPS stimulation. Heward James et al. (117) identified the expression of six eRNAs induced by human monocyte THP1 cells after the LPS activation of the intrinsic immune response, including MARCKS-eRNA, ACSL1-eRNA, AZIN1-eRNA, TNFSF8-eRNA. SLC30A4-eRNA and SOCS3-eRNA. Moreover, the intracellular signaling pathways, such as NFκB and mitogen-activated protein kinase (MAPK), were demonstrated to regulate extracellular kinase 1/2 and p38, which could promote inflammation-associated eRNA expression (117). IIott Nicholas et al. (118) identified 76 differentially expressed eRNAs in primary human monocytes stimulated by LPS and found that the knockdown of the pro-inflammatory TFs, NFκB-mediated IL1b eRNA and IL1b-RBT46 eRNA, attenuated LPS-induced mRNA transcription and pro-inflammatory mediator release, including IL1b and CXCL8. Using reduced representation bisulfite sequencing (RRBS) technology, smoking-associated differentially methylated regions (SM-DMR) was found to up-regulate AHRR mRNA by activating the AHRR enhancer that expressed AHRR eRNA (124). Additionally, smoking-altered methylation and intragenic AHRR enhancer-produced eRNA were found to be necessary prerequisites for monocyte type-specific AHRR transcription (124). Moreover in THP-1 monocytes, hHS-8 was shown to target dCas9-KRAB at the IRF1 binding site to impair IFN-gamma expression on LPS-induced TNF genes and eRNA, thereby affecting monocyte inflammatory immune action (125). Current studies suggest that eRNAs regulate the direction of monocyte function; however, their functional mechanism remains unknown.

#### eRNA in Lymphocyte

As important cellular components of inflammatory immune response, lymphocytes are the main performers of the lymphatic system immune function. They are mainly responsible for fighting external infections and monitoring cellular mutations in the body, and are categorized into B lymphocytes, T lymphocytes and natural killer cells.

##### eRNA in B Lymphocytes

Studies have reported that eRNA transcription during B lymphocyte growth and development is closely associated with large-scale changes in DNA cytosine modifications (126). Moreover, Brazao et al. (127) identified 73 PAX5-dependent eRNAs near protein-coding genes in B-ALL cells, such as LNCGme00432, LNCGme0034 and LNCGme00345, that were the downstream genes of B-cell lymphoma 11a (Bcl11a), whose dysfunction may lead to the malignant development of B cells. Furthermore, Saintamand et al. (128) reported that LPS-induced stimulation *in vitro*, whereas 3RR eRNA deletion reduced transcription and disrupted downstream CH basal transcription, which affected the resting and active states of B cells.

Many eRNA studies have been conducted in the classical B lymphocyte line (GM12878); however, only a few studies have explored the direct effects of enhancer transcription and eRNAs on B lymphocytes. Kim et al. (119) confirmed that enhancers dynamically modulate the transcriptional activities of eRNAs and pre-mRNAs in B-lymphoblasts. Furthermore, the knockdown of TNFSF10-related eRNAs leads to selective regulation in interferon-induced apoptosis, indicating that eRNAs are necessary for target gene induction and can be potential target genes *via* transcriptional reprogramming (119). Katla et al. (129) identified quantitative trait loci associated with eRNA expression and direction-dependent enrichment at enhancer regions in human lymphoblastoid cell lines using capped-nascent-RNA sequencing. These loci were correlated with gene expression, defined central TF binding regions and flanking eRNA initiation cores, which are important indicators of non-coding regulatory variants. Therefore, B lymphocytes have important functionality in enhancer transcription and eRNAs, which affect the growth, differentiation and malignant progression of B lymphocytes; however, further extensive studies are required.

##### eRNA in T Lymphocytes

The exploration of acute T lymphocytic leukaemia leads to unrevealing the relationship between eRNAs and T lymphocytes, confirming the presence of a large number of eRNAs in T-ALL cells (130, 131). Ets1 is a sequence-specific transcription factor that plays an important role during hematopoiesis, and is essential for the transition of CD4−/CD8− double negative (DN) to CD4+/CD8+ double positive (DP) thymocytes. During early T cell differentiation, eRNA shows a DN to DP transition pattern and the Ets1 pattern in DP transition, similar to the RNAPII pattern, suggesting that eRNAs act as active regulatory elements that regulate Ets1 on nucleosome occupancy and enhancer activity to influence T cell differentiation (132). Trimarch et al. (133) identified the first functional eRNA (LUNAR1) in T-ALL cells in 2014, which enhances IGF1R mRNA expression and maintains the IGF1 signaling pathway *via* Notch signaling, thereby maintaining T-Acute lymphoblastic leukaemia (ALL) malignant progression. Subsequently, Tan et al. (102) identified the second functional eRNA (XLOC_005968) in T-ALL cells, namely ARIEL, which recruits intermediary proteins to the ARID5B enhancer, promotes enhancer-promoter interaction and activates ARID5B expression, and thereby positively regulating TAL1-induced transcription and MYC oncogene expression, to accelerate T cells malignant progression.

Recently, some studies have explored the effect of eRNAs on the generation and differentiation of T cells. Hertweck et al. (96) revealed that eRNAs transcribed by T-bet SE were mostly Th1-specific in T cells. In Th1 cells, eRNAs of IFNG upstream SEs were transcribed, while the downstream enhancer exhibited lower levels of P-TEFb occupancy and eRNA production, which affects Th cell differentiation. Luke et al. (125) found that activated CD4+ T cell increased hHS-8, TNF and LTA promoter H3K27 acetylation and nuclease sensitivity while synergistically inducing TNF, LTA and hHS-8 eRNA transcription to regulate TNF mRNA and LTA mRNA, affecting T cell pathology.

##### eRNA in Natural Killer Cells

To date, no studies have explored the effects of eRNAs on natural killer (NK) cells generation, development and differentiation. Only one study has shown that the H3K27me3 histone demethylase UTX controls specific gene expression programs during development of natural killer T cells through demethylase activity-dependent manner (134). We believe that enhancer transcription events and eRNAs have a potentially important role in NK cells as potential therapeutic targets and prognostic biomarkers in inflammatory immune diseases and tumors.

### Inflammatory Function of eRNA in Non-Inflammatory Immune Cells

The inflammatory immune functions of eRNAs are not limited to inflammatory immune cells. The recent studies showed that non-immune inflammatory cells have inflammatory changes and altered cellular states under eRNA regulation (135–138). Isidore et al. (136) found that VEGFA-eRNA5 and VEGFC-eRNA3 in endothelial cells affect angiogenesis and lymphangiogenesis by regulating endogenous transcription and VEGFA and VEGFC expression. Additionally, eRNAs were found to be well correlated with VEGF expression across cell types and in response to hypoxic stimuli using GRO-Seq. Zhou et al. (138), in the same year, revealed that lncRNA-MAP3K affects inflammatory factor (e.g. ICAM-1, E-selectin and MCP-1) expression, reduces monocyte-endothelial cell adhesion and decreases TNF-α, IL-1β and COX2 expression in macrophages through the p38 MAPK signaling pathway and MAP3K4 cis-modulation, which ultimately regulates vascular inflammation. Despite that the overlapping features of lncRNA-MAP3K with 1D-eRNA, strong evidence supporting lncRNA-MAP3K4 as an eRNA that is transcribed from the MAP3K4 enhancer region is lacking (138). Conversely, glucocorticoids have been shown to induce KLF9 expression in lung epithelial cells, with the identification of three common glucocorticoid receptor binding sites that influenced KLF9 mRNA and protein expression levels by generating eRNAs, which ultimately affected glucocorticoid-induced anti-inflammatory effects (135). Additionally, Yukako et al. (137) showed that Nrp2-eRNA, Dcstamp-eRNA and Nfatc1-eRNAs could regulate the corresponding promoters to control gene expression, thereby positively regulating osteoclast differentiation to maintain bone resorption function. These studies, therefore, indicate the potential inflammatory role of eRNAs in non-inflammatory immune cells. However, further studies are required to explore their specific mechanisms and functions.

### eRNA in Inflammatory Immune Diseases

NFκB is an important gene regulator involved in innate and adaptive immune responses as well as survival and proliferation of certain cell types (139), and eRNAs have been shown to be involved in the regulation of inflammatory transcriptional networks (10, 118, 140). Studies have shown that NFκB contributes to the synthesis of inflammatory gene-associated enhanced eRNAs, which further enhances transcription by looping enhancers and promoters or by recruiting RNA polymerase II to the promoter, creating a transcription-mediated multilevel cascade regulating transcription (10, 13, 81, 91). Therefore, this paper further summarizes the potential functions and mechanisms of eRNAs in various inflammatory immune-related diseases (**Table 2**).

**Table 2:** List of eRNAs involved in inflammatory immune diseases.

Disease	Affected cell	Enhancer or SE	eRNA	Regulated gene	Mechanism	Function	Reference
**Gastritis**	Gastric adenocarcinoma cells	IL1 enhancer	IL1 eRNA	IL1	Mediation of NF-kB	Influence inflammatory gene expression	(92)
	Gastric epithelial cells	BIRC3 enhancer	BIRC3 eRNA	BIRC3 cIAP2	Induction of caspase-3 activation	Improve apoptosis resistance in gastric epithelial cells	(94)
**Inflammatory bowel disease**	CD14+ cells	IFNG	IFNG-R-49	IL22 IL26	No	Regulate inflammatory factors	(138)
**Systemic Lupus Erythematosus**	Monocyte	Enhancer 2	eRNA157	ADAMDEC1	Mediation of MAP kinase and p300-NFκB	Regulate of SLE-related inflammatory gene expression	(93)
**Autoimmune uveitis**	CD4+ T cells	SEs	Ifng eRNA	Th1 gene	Recruition of media and P-TEFb	Promote cell-specific gene expression	(91)
**Recurrent pregnancy loss**	Trophoblast cells	No	lnc-SLC4A1-1	CXCL8	Recruition of NFκB and CXCL8	Exacerbate inflammatory response	(95)
**Hypoxia**	Cardiomyocytes	No	HERNA1	SMG1 SYT17	Mediation of phosphatidylinositol 3-kinase-related kinases	Regulate of hypoxic progression and metabolism	(141)

#### eRNA in Gastritis

The chronic inflammation and apoptosis resistance associated with Helicobacter pylori infection contributes to gastric disease development, including gastritis and gastric cancer (142). Chen et al. (97) were the first to report that H. pylori stimulated the recruitment of RelA and Brd4 to inflammatory gene-related enhancers and promoters. Following this, IL1A and IL1B eRNA expressions were up-regulated to affect NFκB-dependent inflammatory gene expressions (e.g. IL1); however, JQ1 was found to attenuate the H. pylori-induced eRNA and mRNA synthesis of NFκB-dependent inflammatory gene subpopulations by inhibiting Brd4-related functions, which suppresses inflammatory immune cell proliferation in H. pylori-infected mice (97). Subsequently, Brd4 was shown to de-activate cIAP2 expression by activating BIRC3 eRNA synthesis in H. pylori infection, which in turn inhibited caspase-3 activation, and ultimately inhibiting cell apoptosis. Therefore, the novel role of BIRC3 eRNA in H. pylori-mediated apoptosis resistance was speculated (99).

#### eRNA in Inflammatory Bowel Disease

Inflammatory bowel disease (IBD), a chronic inflammatory intestinal immune disease, has an unknown molecular pathology. Studies have reported significant differences in eRNA expression levels among multiple chemokine gene-related enhancer regions (including CXCL1-3, CXCL5-6 and CXCL8), which are all up-regulated in IBD, Crohn’s disease (CD), ulcerative colitis (UC) and controls using CAGE and qPCR (143). Baillie et al. (90) used the human monocyte-derived macrophage as a model to explore the genetic aetiology of IBD. They found that transient eRNA transcripts at multiple loci precede promoter-associated transcripts under LPS induction, which affects the adaptation of monocytes to the gastrointestinal mucosal environment, thus leading to IBD. Additionally, Aune et al. (141) revealed a significant association between eRNA genomic location and disease-specific genetic polymorphisms in IBD. It also suggested that the transcription site of the IFNG-R-49 eRNA was more than 100 kb away from the IL26 and IL22 genes, which are consistent with the biological functions exhibited by the eRNA.

#### eRNA in Rheumatoid Arthritis

Rheumatoid arthritis (RA) is a systemic autoimmune disease characterized by chronic synovial inflammation. Various studies have identified the basic leucine zipper transcription factor 2 (BACH2) protein to be a key transcription factor of Treg cells for immune homeostasis. It regulates the expression of various cytokines, including INF-γ and cytokine receptors, whose mutations are associated with RA development and progression (144). BACH2 proteins have been reported to negatively regulate eRNA expression; however, eRNA types and potential mechanisms remain unclear (144). Many disease-associated variants in non-coding regions act by affecting gene transcription and are known as eQTL (145). Studies found that eQTLs were involved in eRNA transcriptional regulation and produced cell type-specific effects, such as STAT6 eQTL up-regulation in patients with RA upregulated inflammatory cytokine production (145–147). Unfortunately, there are no studies that specifically identify which eRNAs influence the occurrence and progression of RA by which mechanisms.

#### eRNA in Other Inflammatory Immune Diseases

Many studies have shown that eRNAs plays a critical role in various inflammatory diseases. The close correlation between ADAMDEC1 and ADAM28 in systemic lupus erythematosus (SLE) regulates the disease inflammatory process. Shi et al. (98) found that monocyte ADAMDEC1 over-expression in patients with SLE was induced by the stimulation of pro-inflammatory cytokines, moreover, under LPS stimulation, the binding of the p300-NFκB complex to enhancer 2 generates eRNA157 that promotes p300 activation, leading to an increase in H3K27ac at the enhancer and promoter region, thus, affecting the regulation of ADAMDEC1 mRNA and SLE-related inflammatory gene expression. Hertweck et al. (96) found that in a mouse model of autoimmune uveitis, T-bet allows mediator and P-TEFb recruitment in the form of SE by extending the SE-generating Ifng eRNA. Therefore, Th1 expression is activated, which triggers IFN-gamma-mediated CD4+ T cells to promote immunoretinol-like binding proteins in the retina. Additionally, flavanols and JQ1 can inhibit SE and its products to down-regulate related gene expression (such as Ifng, Tnf, Fasl, II18r1 and Ctla4), and ultimately decrease the severity of the disease. Furthermore, Huang et al. (100) first found that lnc-SLC4A1-1 was retained in the nucleus as an eRNA and facilitated TF NF-κB binding to CXCL8 promoter region, leading to an increase in H3K27ac in the CXCL8 promoter and subsequently elevated CXCL8 expression. CXCL8 activation is exacerbated by the induction of TNF-α and IL-1β inflammatory response in trophoblast cells, resulting in unexplained recurrent pregnancy loss. It was also found that hypoxia-inducible enhanced RNA 1 (HERNA1) is produced by direct hypoxia-inducible factor 1α binding to the hypoxia response element of histone h3-lysine27. Synaptotagmin XVII, membrane transport proteins, Ca2+ sensing protein and SMG1 are also encoded by phosphatidylinositol 3-kinase-related kinase, thereby regulating immune disease progression, metabolism and contraction (148). Spurlock et al. (149) found that whole blood eRNA expression data effectively classified and differentiated patients with multiple sclerosis from those with other inflammatory and non-inflammatory neurological diseases.

### Inflammatory Function of eRNA in Cancers

Cancer is currently a significant cause of death worldwide and it is a heterogeneous disease controlled by genetic and epigenetic alterations and transcriptional dysregulation (150). Numerous studies have been conducted to show that the abnormal expression levels of eRNAs, as an excellent marker of active enhancers and genes, are associated with dysregulation of enhancer transcription and gene expression in tumors (34, 151–153). Santanu Adhikary (33), Joo-Hyung Lee (34) and Zhao Zhang et al. (154) have each summarized in detail the potential functions, regulatory mechanisms and clinical therapeutic implications of eRNAs in cancer. Nevertheless, this study will focus on the potential functions and mechanisms of eRNAs in tumorigenesis development on its immune microenvironment and related inflammation (**Table 3**).

**Table 3:** List of eRNAs involved in inflammatory effects in tumors.

Cancer	Affected cell	Enhancer or SE	eRNA	Regulated gene	Mechanism	Function	Reference
**Pre-B ALL**	Pre-B ALL cell GREAT	No	eRNA	ICOSLG, IRF4, MSA1	Down-regulate migration, proliferation and apoptosis gene	Promotes malignant progression	(151)
**Pre-B ALL**	Pre-B ALL cell	CD19+/CD20+ spectrum SE	eRNA	ETV6-RUNX1	Interference with B-cell signaling and adhesion signaling	Disrupt normal B lymphopoiesis	(152)
**T ALL**	Leukemia cell	ARID5B enhancer	ARIEL eRNA	ARID5B MYC	Recruition of intermediary complexes and Stable E-P rings	Involve in T-cell leukemia formation	(97)
**KSHV-infected primary** **effusion lymphoma**	TRExBCBL1 cell	MYC SE	MYC eRNA (e486, e507, e530)	MYC	Alteration of host epigenome status	Promote viral lysis and replication	(153)
**Chronic granulocytic leukemi**	Leukemia cell	Hmrhl enhancer	Hmrhl eRNA	phkb	No	Regulate positively phkb genes	(154)
**MLL rearranged leukemia**	MLL leukemia cells	Enhancer	SEELA eRNA	SERINC2	Promotion of histone recognition	Regulate tumor metabolism	(155)
**Chronic myeloid leukemia**	Leukemia cell	VEGFA157 enhancer	VEGFA157 eRNA	VEGFA	Selective splicing of target genes	Promote CML pathology	(156)
**Gastric cancer**	Gastric epithelium	BIRC3 enhancer	BIRC3 eRNA	BIRC3 cIAP2	Inhibition of caspase-3 activation	Enhance apoptosis resistance in gastric epithelial cells	(92, 94)
**Colorectal cancer**	CRC cell	Carcinogenic SE	eRNA	IL-20RA	Elevated expression of cell proliferation and immune evasion genes	Modulate carcinogenic and immune pathways	(157)

#### eRNA in Haematologic Malignancies

The relevant functions and mechanisms of eRNAs in inflammatory immune cells have been previously summarized, along with the exploration of the potential functions and mechanisms of eRNAs in immune hematopoietic system-related tumors. Almamun et al. (155) were the first to report that the aberrant methylation of enhancer regions was associated with the altered expression of neighboring genes involved in cell cycle processes, lymphocyte activation and apoptosis in pre-B ALL. Further studies have suggested an overall downregulation of eRNA transcripts in patients with pre-B ALL, which may affect the downregulation of target genes (such as ICOSLG, IRF4 and MSA1) in B-cell migration, proliferation and apoptosis (156). Further, Teppo et al. (157) used eRNA quantification to elucidate the aberrant transcriptional activity downstream of fusion TFs and demonstrated that the ETV6-RUNX1 axis regulates cell adhesion and transmembrane signaling pathways, which ultimately disrupts normal B lymphangiogenesis. Tan et al. (102) were the first to demonstrate the involvement of lncRNA in the regulation of TAL1-induced T-ALL oncogenic regulatory program. They also showed that XLOC_005968, the ARIEL eRNA, is oncogenic and positively regulated by ARID5B and MYC oncogene expression in T-ALL cells by recruiting mediator complexes and promoting ARID5B enhancer-promoter interactions (102). Additionally, Kaposi’s sarcoma-associated herpesvirus (KSHV), a human tumorigenic γ-2 herpesvirus, is the pathogen responsible for Kaposi’s sarcoma and primary effusion lymphoma. It is proposed that KSHV reactivation decreases MYC gene expression by downregulating MYC eRNA expression and enhancer activity, and shRNA-mediated and vIRF4-mediated cIRF4 suppression, which promotes lytic replication (158).

In leukaemia, a novel Hmrhl eRNA was shown to be highly upregulated in chronic granulocytic leukaemia (CML) cells and positively regulate its host gene phkb expression (159). Additionally, Fang et al. (160) found that eRNAs such as SEELA are widely activated in mixed-lineage leukaemia and demonstrated that SEELA directly binds to amino acid K31 of histone 4 and mediates the cis-activated transcription of the neighboring oncogene serine incorporate 2, which regulates oncogene transcription and tumour metabolism (sphingolipid synthesis) to influence leukaemia progression. The demethylation of vascular endothelial growth factor A (VEGFA) enhancer in CML promotes the overexpression of cancer signature genes. A study by Dahan showed that VEGFA+157 eRNA regulates its selective splicing, which affects CML cell proliferation by increasing RNAPII elongation *via* CCNT2 (161).

#### eRNA in Other Cancers

To date, eRNA inflammatory immune-related functions are only marginally studied in solid tumors. H. pylori infection is a major cause of gastric cancer, and its pathogenicity is associated with chronic inflammation induction and apoptosis resistance. The inflammatory immune role of eRNA in tumors was first reported by Chen et al. (99), demonstrating that H. pylori stimulate bromodomain-containing factor Brd4 recruitment to the BIRC3 enhancer, which promotes BIRC3 eRNA synthesis and cIAP2 expression, which inhibits caspase-3 activation and enhances apoptosis resistance in gastric epithelial cells. Additionally, some studies have suggested the presence of oncogenic SEs in colorectal cancer and confirmed their involvement in regulating oncogenic and immune pathways in colorectal cancer by modulating IL-20RA expression, affecting cell proliferation and immune evasion-related gene ecpression (162). Pancreatitis accelerates Kras mutation-driven tumorigenesis in mice, which is mostly found in pancreatic ductal adenocarcinoma (163). Li et al. (163) reported that under inflammatory stimulation, KrasG12D mutation targets a transient enhancer network driving proto-oncogene transcription and provides a sustained Kras-dependent oncogenic program to drive tumour tissue-specific progression. However, they did not explore the specific mechanism and types of enhancers and eRNAs.

The rapidly rising development of bioinformatic technologies provides novel means and directions to study the role of eRNA in inflammatory immunity in tumors. Various bioinformatics analyses have initially suggested that eRNA expression levels are significantly correlated with malignancy prognosis and can affect the tumour immune microenvironment (164–170). Furthermore, Xiao et al. (164) found that LINC02257 eRNA was significantly associated with cancer survival and immunotherapy-related indicators (e.g. tumour microenvironment, tumour mutational load and microsatellite instability). A study by Wang et al. (165) identified WAKMAR2 eRNA as a key candidate biomarker in invasive breast cancer, which may influence the tumour microenvironment by regulating the relevant immune-related genes, such as RAC2, IL27RA, IGLV1-51, IGHD, IGHA1 and FABP7. AC003092.1 eRNA and glioblastoma multiforme (GBM) overall survival were significantly correlated, and AC003092.1 eRNA was shown to be associated with the immunosuppressive microenvironment of GBM using single gene set enrichment analysis and CIBERSORTx system analysis (166). Using similar bioinformatic techniques, several studies have suggested that functional FOXO6-eRNA can regulate FOXO6 expression to influence EGFR and SOX2 expression and function in lung cancer progression (167); furthermore, the aberrant expression of LINC00987/A2M axis was closely associated with immune cell infiltration in lung adenocarcinoma (168). AC007255.1 in esophageal cancer was reported to be closely associated with tumour immune response and neutrophil activation (169). Additionally, AP001056.1 was shown to be enriched in the biological function analysis of head and neck squamous cell carcinoma, mainly in immune system processes (170). However, bioinformatic analyses can only tentatively suggest the potential role of relevant eRNAs in tumors. Therefore, further basic research and clinical trials are required to validate these results.

### Clinical Perspectives of Enhancer Inhibitors Affecting eRNA Production

**Figure 4:**
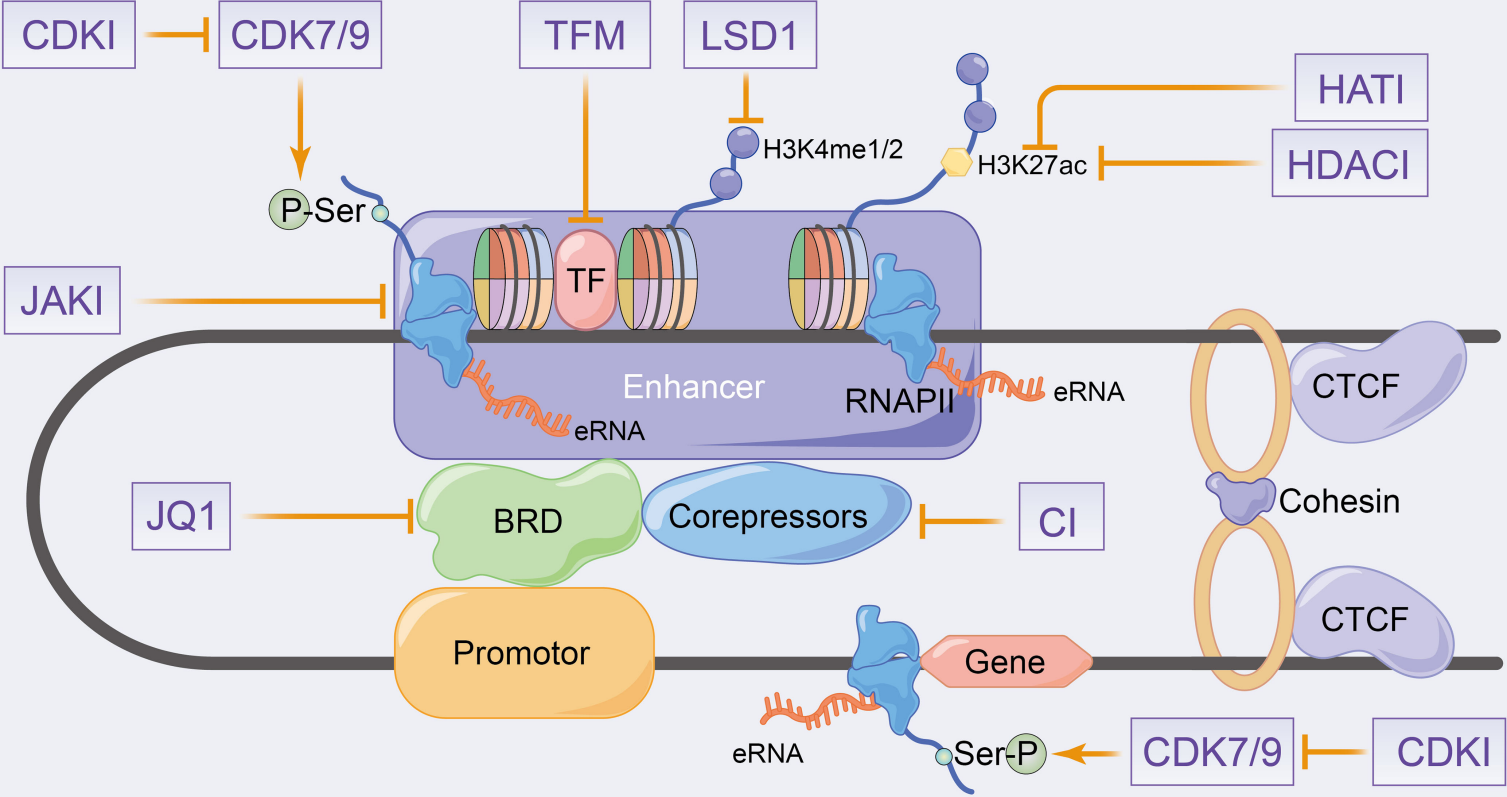
Schematic representation of putative therapeutic targets to enhancer transcription events and eRNA landscapes. CDKI, cyclin-dependent kinase inhibitors; HATI, histone acetyltransferase inhibitors; HDACI, histone deacetylase inhibitors; LSD1, lysine-specific demethylase 1; JAKI, janus kinase inhibitors; TFM, transcription factors inhibitors; CI, co-repressors inhibitors.

Many eRNAs are significantly differentially expressed in tumour tissues compared with paracancerous tissues (151, 154), which is consistent with the results of enhancer overactivation in cancer (171–174). Therefore, eRNAs can be considered as potential targets to overcome enhancer activation in cancer therapy. eRNAs have high specificity across tissues and tumours (41, 151, 154), with the antisense oligomer-based targeting of specific eRNAs effectively inhibiting target genes and tumour growth without theoretically affecting other unrelated tissues (49, 82, 102, 154, 175, 176). Therefore, eRNAs can be used as effective and highly precise therapeutic targets in cancer therapy. Regrettably, no relevant eRNA-targeted drugs are currently on the market or in clinical trials. In this section, we will therefore concentrate mainly on enhancer inhibitors affecting eRNA production to elucidate their potential therapeutic effects on inflammatory immune diseases and even cancer. The eRNA-related agents in cancer have been previously reviewed, including bromodomain and extra-terminal (BET) inhibitors, cyclin-dependent kinases (CDKs), HAT inhibitors and HDAC inhibitors (33, 34, 177). Since inhibitors that fully target eRNAs in inflammatory immune-related diseases have been rarely studied, the combined inhibitors of eRNAs and active enhancers, which produce eRNAs, have been summarized along with their therapeutic potential in inflammatory immune diseases in **Figure 4**.

#### ET Inhibitors in eRNA

Estradiol cypionate (ET) inhibitors, such as JQ1 and I-BET762, can recognize acetylated histones by interfering with the BET family proteins BRD2, BRD3, BRD4 and BRDT (178). Thus, targeting this protein class can promote LPS-induced inflammatory gene expression in macrophages and significantly improve survival, as observed in an *in vivo* sepsis model (178, 179). JQ1 primarily targets BRD4 to inhibit TNF-α or IL-1β-induced inflammatory cytokines expression and reduces enhancer-mediated inflammatory responses and diseases (180–183) (**Figure 4**). H. pylori stimulates NFκB-dependent BRD4 to enhance inflammatory gene-related eRNA synthesis, similarly, JQ1 inhibits BRD4 to reduce eRNA synthesis and inhibits RNAPII recruitment that is induced by BRD4 interaction with RelA. Additionally, JQ1 has been reported to inhibit inflammatory gene expression and inflammatory immune cell proliferation in H. pylori-infected mice (97). Arnulf et al. (96) demonstrated that the treatment of Th1 cells with JQ1 and xanthinol resulted in the inhibition of P-TEFb, which produced a significant reduction in eRNA levels, including Ifng eRNA. This also promoted reduction in SE-related Th1 gene expression (e.g. Ifng, Tnf, Fasl, IL18 and Ctla4), which caused disease remission in autoimmune uveitis mice. Angela et al. (158) reported that the associated MYC eRNAs (including e486, e507 and e530) was significantly decreased after the JQ1 treatment of KSHV-infected primary effusion lymphomas, which inhibited MYC expression and KSHV cleavage gene expression induction. Additionally, a decrease in MYC mRNA was found on the knockdown of the corresponding eRNA (158). JQ1 was demonstrated to decrease the transcriptionally activated eRNAs of SEs, causing the down-regulation of IL-20RA expression and inhibition of growth, metastasis and immune escape in colorectal cancer (162). Therefore, JQ1 regulates enhancer transcription and eRNA synthesis by affecting the mediator complex, which regulates the downstream target genes to modulate the disease process.

#### CDK Inhibitors in eRNA

CDK7 is a component of the transcription initiation factor IIH (TFIIH) in the GTF complex that regulates enhancer and target gene transcription by phosphorylating Ser5 and Ser7 on RNAPII (184). Additionally, CDK7 activates and phosphorylates the P-TEFb catalytic subunit of CDK9, which phosphorylates Ser2 in the RNAPII CTD to control transcriptional elongation and termination (184) (**Figure 4**). Currently, transcriptional CDKs are considered potent targets for cancer therapy (185–189), and recently, some studies have also evaluated the role of CDK inhibitors in inflammatory-immune diseases (190–192). The therapeutic role of CDK7 in haematologic malignancies has been widely reported that demonstrate the deletion of oncogenic transcription factors, such as RUNX1, by small-molecule CDK inhibitors in acute T-lymphoblastic leukaemia (189). Moreover, SY-1365, a highly selective CDK7 inhibitor, is currently used in clinical trials in patients with ovarian and breast cancer (187). These studies have confirmed that CDK7 inhibitors reduce enhancer-associated oncogene expression by modulating eRNA expression levels. CDK inhibitors such as THZ1, NVP-2 and THZ531 that inhibit CDK7, CDK9, CDK12 and CDK13, respectively, have been shown to downregulate SE-related oncogene expression and promote DNA damage response gene loss in chordoma and acute T-lymphoblastic leukaemia cells, respectively (185, 186, 188). Furthermore, the blocking of CDK7 has been reported to regulate the onset and intensity of immune-inflammatory responses by activating the tumour immune response and regulating granulocyte apoptosis and cytokine secretion (193, 194). Recently, transcriptional CDKs have been speculated to play an important role in pro-inflammatory gene expression (190), with Wei et al. to be the first to demonstrate in the cytokine release syndrome (CRS) that the CDK7 covalent inhibitor THZ1 downregulates inflammatory gene transcription in macrophages after preferential target inhibition associated with SEs, such as STAT1 and IL1, decreases cytokine release, alleviates the hyperinflammatory state and rescues lethal CRS mice (191).

#### Other Inhibitors in eRNA

Furthermore, enhancer transcription analyses and eRNA generation processes revealed that TF modulators, HAT inhibitors and HDAC inhibitors influence eRNA and mRNA production by modulating enhancer epigenetic characteristics (177) (**Figure 4**). HATs, such as p300-CBP, enable histone tail acetylation modifications (195), whereas polycomb repressive complex (PRC) mediates histone methylation. The drugs targeting PRC inhibit leukaemia-associated enhancer transcription, control pro-apoptotic B cell lymphoma-2 like 11 and mediate apoptosis in breast cancer cells (196, 197). HDACs mediate histone deacetylation, with numerous studies demonstrating the effect of HDAC inhibitors on enhancer landscape in various cancer types (197–199). Lysine-specific demethylase 1 (LSD1) was identified as a selective mediator of H3K4 demethylation, with LSD1 inhibitors affecting the progression of acute myeloid leukaemia by disrupting the enhancer with the SNAG structural domain transcriptional repressor GFI1 (200). Additionally, LSD1 inhibitors have been shown to affect enhancer activity in various tumors, such as androgen receptor function in prostate cancer (201, 202) and ERα activity in breast cancer (203). However, relevant studies exploring the potential therapeutic efficacy of HAT inhibitors and HDAC inhibitors in inflammatory immunity as along with their mechanisms and function on eRNA are lacking.

Notably, a study by Huang et al. demonstrated that corepressor recruitment (GPS2 and SMRT) is a genome-wide signature of inflammatory immune enhancers, which antagonizes eRNA transcription and CBP-mediated H3K27 acetylation. This reverses subinflammation and insulin resistance by providing targeted eRNA therapeutics for immunometabolic diseases (123). A Janus kinase inhibitor (tofacitinib) has been shown to block cytokine signaling in T cells, thereby affecting RA risk gene expressions and SE structure, which ultimately targets autoimmune diseases (144). Therefore, targeted small molecule drugs that affect enhancer transcription and eRNAs can be considered as potential therapeutic targets for inflammatory immune-related diseases and tumors. Although enhancer inhibitors have great potential in diseases with inflammation immune alterations, further clinical trials are needed to validate. In addition, small molecule inhibitors specifically targeting eRNA still have great potential for exploration and development.

## Discussion

Rapid advances in sequencing and microscope technologies suggest the potential contribution of enhancer transcription and eRNAs in inflammatory immune-altered diseases. eRNAs have been considered to have induced a breakthrough in the field of targeted therapy, spurring various studies centered on transcriptional precision and complexity. The activation modes of enhancers and SEs and regulatory mechanisms of enhancer transcription and eRNAs on target genes have been extensively analysed in this review. However, owing to the limited understanding of enhancer transcription and eRNA biology, multiple questions remain to be addressed: 1) What are the basic features of enhancer-promoter communication, interdependence and base coexistence sequence? 2) How does enhancer transcription regulate eRNA and mRNA expression? 3) How does eRNA activate paired-promoter gene transcription? 4) How do specific structures (e.g. molecular clouds and condensates) formed by eRNA regulate the enhancer and promoter gene and affect related diseases? 5) How does the eRNA epigenetic modification affect the enhancer and promoter gene transcription?

Although studies on enhancer transcription and eRNAs are limited to the fields of cancer development and differentiation, recent studies demonstrate that both enhancer transcription activation and eRNA expression are preferentially expressed over inflammatory immune-related genes under LPS induction. This suggests a potential regulatory mechanism between eRNAs and inflammatory immune genes, which alters inflammatory immune responses in diseases. This review summarizes that eRNA expression levels in inflammatory and non-inflammatory immune cells are significantly correlated with inflammatory gene expression in response to inflammatory stimuli, leading to a rapid transition from a quiescent to an inflammatory transcriptional program, thus affecting the development and differentiation of associated immune and non-immune cells. Notably, the inflammation-associated NFκB signaling pathway contributes to inflammation-associated eRNA synthesis and positively regulates enhancer transcription to form a multi-cascade regulatory transcription that affects various inflammatory immune diseases, such as gastritis, SLE and inflammatory bowel disease. Currently, the inflammatory immunomodulatory role of eRNAs in tumors is limited to hematopoietic malignancies, while studies in substantive tumors are scarce. Consequently, the enhancer transcription processes and eRNAs have been speculated to not only affect the development and differentiation of inflammatory immune cells (monocyte-macrophage and lymphocytes predominantly) but also lead to the alteration in inflammatory-immune responses in various diseases, including tumors. However, the specific mechanisms or signaling pathways by which eRNAs affect cells and diseases remain unclear. However, bioinformatic analyses have made it possible to identify and screen functional eRNAs associated with inflammatory immunity and utilize them as a basis for extensive functional, mechanistic and therapeutic exploration.

Significant advances have been made in the treatment of inflammatory immune diseases and tumors using small-molecule drugs targeting enhancer transcription processes, which provide novel therapeutic directions and tools for diseases with inflammatory immune alterations and their drug resistance. However, further studies are required as the regulation of downstream target genes by eRNAs and the instability and dynamics of eRNA have been scarcely explored. Currently, no relevant studies have explored briefly the efficacy of small-molecule drugs that directly target eRNA on lesioned cells or diseases. However, some RNA-based inhibitors such as locked nucleic acid antisense oligonucleotides (LNA ASOs) have been to identified to have significant efficacy in targeting lncRNAs for silencing, which predicts that LNA ASO may be a potential targeting inhibitor in the eRNA field. Nevertheless, eRNA-associated protein chaperones can be identified using genomics and proteomics, and structure analyses can help in designing small-molecule regulators that specifically target eRNA protein chaperones. Additionally, further studies considering altered eRNA epigenetic modifications (e.g. 5-cytosine methylation, n6-adenosine methylation) and eRNA interactions in higher-order chromatin organization as potential eRNA targets are required. Therefore, this review aims to highlight the usefulness of eRNA as an effective potential therapeutic target and prognostic biomarker for inflammatory immune diseases and tumors with inflammatory immune alterations. However, further studies are required to confirm the functions and regulatory mechanisms of eRNAs in inflammatory-immune alterations and explore the potential therapeutic effects of relevant eRNA small molecule inhibitors in diseases with inflammatory-immune alterations.

## Author Contributions

Overview concept: LW and BX. Literature data collection: LW and YH. Literature analysis: LW, YH, MC, and BX. Funding acquisition: YM, MC, and BX. Software and Visualization: LW. Writing-original draft: LW. Writing-review and editing: LW, WL, YH, MC, and BX.

## Funding

National Natural Science Foundation of China (No. 81872089, 81370849, 81672551); Six talent peaks project in Jiangsu Province; Jiangsu Provincial Medical Innovation Team (CXTDA2017025); The National Key Research and Development Program of China (SQ2017YFSF090096); Innovative Team of Jiangsu Provincial (2017ZXKJQWO7).

## Conflict of Interest

The authors declare that the research was conducted in the absence of any commercial or financial relationships that could be construed as a potential conflict of interest.

## Publisher’s Note

All claims expressed in this article are solely those of the authors and do not necessarily represent those of their affiliated organizations, or those of the publisher, the editors and the reviewers. Any product that may be evaluated in this article, or claim that may be made by its manufacturer, is not guaranteed or endorsed by the publisher.
